# Cyanidin Attenuates Methylglyoxal-Induced Oxidative Stress and Apoptosis in INS-1 Pancreatic β-Cells by Increasing Glyoxalase-1 Activity

**DOI:** 10.3390/nu12051319

**Published:** 2020-05-06

**Authors:** Tanyawan Suantawee, Thavaree Thilavech, Henrique Cheng, Sirichai Adisakwattana

**Affiliations:** 1 Program in Biomedical Sciences, Graduate School, Chulalongkorn University, Bangkok 10330, Thailand; Tanyawan.s@chula.ac.th; 2 Phytochemical and Functional Food Research Unit for Clinical Nutrition, Department of Nutrition and Dietetics, Faculty of Allied Health Sciences, Chulalongkorn University, Bangkok 10330, Thailand; 3 Department of Food Chemistry, Faculty of Pharmacy, Mahidol University, Bangkok 10400, Thailand; Thavaree.thi@mahidol.edu; 4 Department of Comparative Biomedical Sciences, School of Veterinary Medicine, Louisiana State University, Baton Rouge, LA 70803, USA; hcheng@lsu.edu

**Keywords:** cyanidin, methylglyoxal, glyoxalase-1, apoptosis, reactive oxygen species, pancreatic β-cells

## Abstract

Recently, the mechanisms responsible for anti-glycation activity of cyanidin and its derivatives on the inhibition of methylglyoxal (MG)-induced protein glycation and advanced glycation-end products (AGEs) as well as oxidative DNA damage were reported. In this study, we investigated the protective effect of cyanidin against MG-induced oxidative stress and apoptosis in rat INS-1 pancreatic β-cells. Exposure of cells to cytotoxic levels of MG (500 µM) for 12 h caused a significant reduction in cell viability. However, the pretreatment of cells with cyanidin alone (6.25–100 μM) for 12 h, or cotreatment of cells with cyanidin (3.13–100 μM) and MG, protected against cell cytotoxicity. In the cotreatment condition, cyanidin (33.3 and 100 μM) also decreased MG-induced apoptosis as determined by caspase-3 activity. Furthermore, INS-1 cells treated with MG increased the generation of reactive oxygen species (ROS) during a 6 h exposure. The MG-induced increase in ROS production was inhibited by cyanidin (33.3 and 100 μM) after 3 h stimulation. Furthermore, MG diminished the activity of glyoxalase 1 (Glo-1) and its gene expression as well as the level of total glutathione. In contrast, cyanidin reversed the inhibitory effect of MG on Glo-1 activity and glutathione levels. Interestingly, cyanidin alone was capable of increasing Glo-1 activity and glutathione levels without affecting Glo-1 mRNA expression. These findings suggest that cyanidin exerts a protective effect against MG-induced oxidative stress and apoptosis in pancreatic β-cells by increasing the activity of Glo-1.

## 1. Introduction

Methylglyoxal (MG) is a reactive dicarbonyl intermediate produced by the fragmentation of triosephosphates glyceraldehyde-3-phosphate (GAP) and dihydroxyacetone phosphate (DHAP) during glycolysis. Several studies revealed that MG causes cell toxicity through oxidative stress- induced apoptosis, increased caspase activity, regulation of reactive oxygen species (ROS) scavenging enzymes, and depletion of the cellular glutathione redox status [1,2]. Most importantly, the concentration of plasma MG is found to be higher in diabetic patients [3]. In addition, MG initiates cellular degeneration by inducing intracellular ROS production and oxidative damages to protein in pancreatic β-cells [4,5]. It has been shown that MG induces cytotoxicity in pancreatic INS-1 cells through activating oxidative stress and further triggering the mitochondrial apoptotic pathway and ER stress-mediated Ire1α-JNK pathway [6]. This abnormal ROS imbalance contributes to mitochondrial dysfunction that affects insulin secretion and insulin sensitivity at target tissues, leading to hyperglycemia. MG reacts with the amino acids of proteins to form chemically stable advanced glycation-end products (AGEs). The accumulation of AGEs is associated with the progression of diabetic microvascular complications such as retinopathy, neuropathy and nephropathy [7]. In mammals, the glyoxalase system is essential for detoxification from MG. In this process, MG is degraded into D-lactate through enzymatic reactions catalyzed by glyoxalase-1 (Glo-1) and glyoxalase-2 (Glo-2), using glutathione as a co-factor. Increasing activity of the glyoxalase system helps reduce the MG-induced modification of proteins and formation of AGEs in the tissues [8]. Because MG has been implicated in the development of diabetes mellitus, naturally occurring flavonoids from dietary sources have gained considerable attention as a potential agent for the reduction in MG-induced toxicity in pancreatic β-cells [9,10,11]. 

Cyanidin, a type of anthocyanidin, is one of the most abundant flavonoids in fruit and vegetables such as grapes, blackberry, blueberry, apples, red onion, and red cabbage [12]. The anti-diabetic and anti-glycation effects of cyanidin are well established [13,14,15]. For instance, it has the ability to inhibit intestinal α-glucosidase and pancreatic α-amylase, the key enzymes for regulating carbohydrate digestion [16]. In a diabetic mouse model, cyanidin treatment slowed disease progression by preserving pancreatic islet architecture and stimulating insulin secretion [17]. Cyanidin also inhibits glucose- and MG-induced protein glycation and advanced glycation-end products in albumin [18]. Previously, cyanidin’s ability to trap MG and inhibit the conversion to AGEs and generation of free radicals-induced DNA damage was reported. Based on these findings, we hypothesize that cyanidin could prevent MG-induced cell apoptosis in pancreatic β-cells. In this study, we investigated for the first time the protective effect of cyanidin on MG-induced apoptosis in pancreatic β-cells by examining its effect on glyoxalase-1 (glo-1) activity and glutathione levels.

## 2. Materials and Methods 

### 2.1. Chemicals

RPMI-1640 medium, fetal bovine serum (FBS), penicillin-streptomycin and trypsin-EDTA were purchased from Gibco (Grand Island, NY, USA). Methylglyoxal (MG), methylthiazolyldiphenyl-tetrazolium bromide (MTT), 2′,7′-dichlorofluorescin diacetate (DCFH-DA), L-glutathione reduced, sulfosalicylic acid (SSA), 5,5′-Dithiobis(2-nitrobenzoic acid) (DTNB), NADPH, and glutathione reductase were purchased from Sigma-Aldrich Co. (St. Louis, MO, USA). The EnzChek^®^ Caspase-3 assay kits and Dead Cell Apoptosis kit with Annexin V FITC and PI were purchased from Invitrogen (Carlsbad, CA, USA). Cyanidin chloride was synthesized from quercetin according to published method [19]. 

### 2.2. Cell Culture

Rat pancreatic β-cells INS-1 were cultured in a RPMI-1640 medium containing 11 mM glucose supplemented with 10% fetal bovine serum (FBS), 2 mM L-glutamine, 1 mM sodium pyruvate, and 50 µM 2-mercaptoethanol [13]. Cells were maintained at 37 °C in a humidified 5% CO_2_ incubator. All experiments were performed with cells between passages 70 and 85. 

### 2.3. INS-1 Cell Treatment

Cells were seeded into a 96-well microplate (1 × 10^5^ cells/well), 12-well microplate (3 × 10^5^ cells/well), or 6-well microplate (6 × 10^5^ cells/well) and allowed to grow for 24 h. Then, cells were treated with MG at the concentration ranging from 10 to 1000 μM for 12–48 h. The concentration of MG showing 60%–80% cell viability was selected to examine the protective effect of cyanidin in INS-1 cells. A concentration-response was obtained for cyanidin by the pretreatment of cells with 3.13–100 μM for 12 h, and then, exposure to 500 μM MG for 12 h or cotreatment with cyanidin and 500 μM MG for 12 h. At the end of the experiments, cell pellets were collected, aliquoted and stored at −20 °C for total protein and mRNA expression determination. In this study, the final concentration of DMSO in the medium was 0.1%.

### 2.4. Cell Viability 

Cell viability was determined using the MTT assay [9]. Briefly, the MTT solution (500 μg/mL) was added to each well and incubated for 4 h at 37 °C. The formazan crystals in each well were dissolved in DMSO. The absorbance was measured at the wavelength of 570 nm and results were expressed as percentage of cell viability related to the control.

### 2.5. Measurement of Intracellular Reactive Oxygen Species (ROS)

The level of oxidative stress was monitored according to published method with minor modification [20]. Following treatments, 10 μM DCFH-DA in 0.1 M phosphate-buffered saline (PBS, pH 7.4) was added, and then, incubated for 30 min at 37 °C in light-protected conditions. Cells were washed twice using 0.1 M PBS (pH 7.4) to remove the redundant DCFH-DA and 0.1 M PBS (pH 7.4) added into each well. The fluorescent intensity was measured at 480 nm excitation wavelength and 530 nm emission wavelengths and results were expressed as the percentage of intracellular ROS production compared to the control.

### 2.6. Caspase-3 Activity

INS-1 cells were lysed with 200 μL lysis buffer and the activity of caspase-3 measured using an EnzChek^®^ Caspase-3 assay kit (Thermofisher, Walham, MA, USA) according to the manufacturer’s instructions. 

### 2.7. Flow Cytometry

Cell apoptosis was determined according to published method with minor modification [21]. Briefly, floating and adherent INS-1 cells (6 × 10^5^) were resuspended in 100 μL of binding buffer and incubated in the dark with both Annexin V and propidium iodide (PI) for 15 min. Then, a minimum of 10,000 cells per sample was examined by FACSCalibur flow cytometer (Becton Dickinson, Mountain View, CA, USA). The percentage of living (negative in both Annexin V-FITC and PI), early apoptotic (positive in Annexin V-FITC and negative in PI), late apoptotic (positive in both Annexin V-FITC and PI), and necrotic cells (positive only in PI) was calculated. Untreated cells were used as controls for double staining.

### 2.8. Glyoxalase 1 Activity

INS-1 cells were lysed with lysis buffer (400 µL) and centrifuged at 2100× *g* at 4 °C for 10 min. The supernatant was stored at −20 °C until glyoxalase 1 (Glo-1) activity assay was performed according to the published method with minor modification [20]. The activity of Glo-1 was measured using the initial rate of S-D-lactoylglutathione formation from hemi-thioacetal. The hemi-thioacetal adduct was obtained by incubating an equimolar mixture of MG (500 µM) and reduced glutathione (2 mM) in 50 mM PBS (pH 6.6) at 37 °C for 10 min. The cell lysates (10 µL) were incubated with the hemi-thioacetal adduct at 25 °C for 1 h in the 96-well UV plate. The absorbance was measured at 240 nm wavelength and subtracted from the baseline. The results were expressed as the percentage of Glo-1 activity compared to controls.

### 2.9. RNA Isolation and Reverse Transcription-Quantitative PCR (RT-qPCR)

RNA was extracted using TRIzol reagent (Invitrogen, Carlsbad, CA, USA) according to the manufacturer’s instructions. The RNA concentration was quantified by a NanoDrop ND1000 UV Visible Spectrophotometer (NanoDrop Technologies, Rockland, DE, USA), and 1 μg of total RNA was reverse transcribed to cDNA using ImProm-II™ Reverse Transcription System from Promega Corporation (Madison, WI, USA) according to the manufacturer’s instructions. The primer sequences were obtained as follows: glyoxalase 1 (Glo 1, forward: 5′-GAAGCCTGATGATGGGAAAA-3′ and reverse: 5′-TCTCAGCATCTCGAATCACG-3′) and glyceroldehyde-3-phosphate dehydrogenase (GAPDH, forward: 5′-CATGAGAAGTATGACAACAGCCT-3′ and reverse: 5′-AGTCCTTCCACGATACCAAAGT-3′) [22]. RT-qPCR was performed with CFX Connect™ Real-Time PCR Detection System (Bio-RAD Laboratories Inc., Irvin, CA, USA) using SsoFast™ Evagreen Supermix SYRB green detection (Bio-RAD) according to the manufacturer’s instructions. All samples were carried out in triplicate. Quantification was normalized to the GAPDH gene as an endogenous internal control.

### 2.10. Measurement of Glutathione

Total glutathione was measured by the enzymatic method using glutathione reductase (GR) [23]. After incubation, cells were collected and washed using 1 mL of ice-cold 0.1 M PBS and centrifuged at 700× *g* for 5 min at 4 °C to remove the supernatant. The cells were lysed with ice-cold extraction buffer (0.1% Triton-X and 0.6% sulfosalicylic acid (SSA) in KPE buffer, pH 7.5) and centrifuged at 3000× *g* for 4 min and the supernatant used for glutathione assay. The GSH standard solutions or the cell lysates (20 µL) were incubated with 120 µL the mixture of equal volume of freshly prepared DTNB (1.5 mg/mL) and glutathione reductase solution (6 units/mL) for 5 min at room temperature. Then, 60 µL of 0.16 mg/mL NADPH was added and read at 412 nm wavelength with kinetics at 1-min intervals for 5 min. Total glutathione in the sample was determine using the standard glutathione calibration curve. The protein concentration in the supernatant was determined with Bradford assay and results expressed as nmol/mg protein.

### 2.11. Statistical Analysis

The results are expressed as mean ± standard error of the mean (S.E.M) from three independent experiments (*n* = 3), each with internal triplicates. Multiple group comparisons were carried out using a one-way analysis of variance (ANOVA), followed by Duncan’s post hoc test (SPSS, Chicago, IL, USA). Statistical significance was established at *p* < 0.05.

## 3. Results

**Figure 1:**
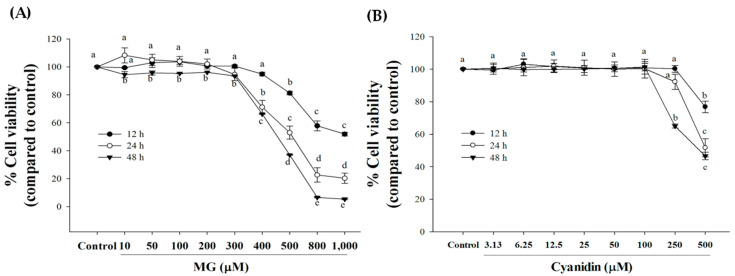
Effect of cyanidin and methylglyoxal (MG) on cell viability during 12–48 h. (**A**) Treatment of cells with MG 500–1000 μM decreased cell viability during a 12–48 h period. (**B**) 100 μM or lower concentrations had no effect on cell viability during a 12–48 h period. Results are presented as mean ± SEM (*n* = 3). The groups that do not share a common letter are significantly different (*p* < 0.05).

Treatment of cells with MG (10–300 µM) for 12–24 h had no effect on cell viability (Figure 1A). A significant decrease in cell viability was observed with MG 400 µM or higher at 12 and 24 h of incubation. Furthermore, the percentage of cell viability with MG (500, 800, and 1000 µM) was 81%, 58%, and 52%, respectively at 12 h of MG exposure. After 24 h incubation, MG (400–1000 µM) significantly decreased INS-1 cell viability (20–71%) compared to controls. In addition, MG (10–1000 µM) exerted cytotoxicity after exposure to cells for 48 h. For further experiments, we selected 500 µM MG and 12 h incubation for further experiments because this condition was able to induce cytotoxicity at the low concentration and short exposure time. 

Concentration-response experiments with cyanidin revealed that 100 μM or lower concentrations had no effect on cell viability (Figure 1B). To test whether cyanidin had a protective effect, cells were incubated with the compound and 500 μM MG for 12 h.

**Figure 2:**
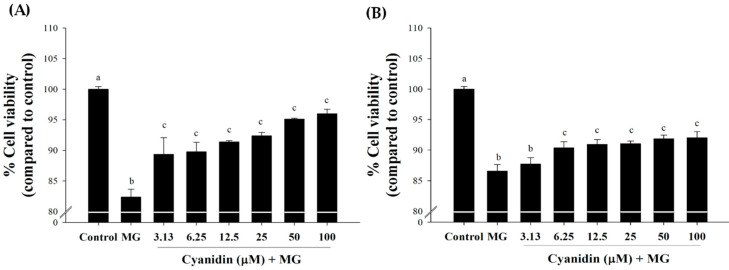
Effect of cyanidin and its treatment with 500 μM methylglyoxal (MG) on cell viability. (**A**) Cotreatment of cyanidin (3.13–100 μM) and MG decreased cytotoxicity during 12 h. (**B**). Pretreatment with cyanidin (6.25–100 μM) for 12 h decreased cytotoxicity during 12 h of MG exposure. Results are presented as mean ± SEM (*n* = 3). The groups that do not share a common letter are significantly different (*p* < 0.05).

The results showed that cotreatment with cyanidin (3.13–100 μM) markedly reduced MG-induced cytotoxicity (Figure 2A). In this condition, cell viability was approximately 90–96% in relative to the control. Pretreatment with cyanidin (6.25–100 μM) for 12 h showed slight cytoprotective effects with ~90% cell viability (Figure 2B). These findings indicate that cyanidin cotreatment had a greater protective effect on MG-induced cytotoxicity than with the pretreatment. Based on these results, we selected the cotreatment of cyanidin with MG for further experiments.

**Figure 3:**
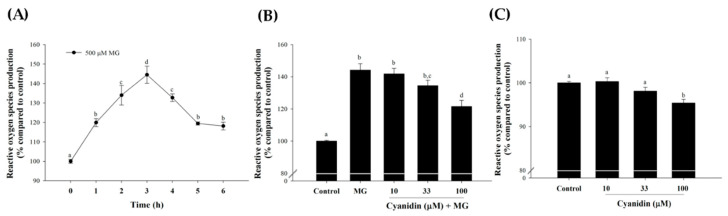
Effect of cotreatment of cyanidin and 500 μM methylglyoxal (MG) for 3 h on the formation of reactive oxygen species (ROS). (**A**) Treatment of cells with MG increased ROS during a 6 h period, with a peak increase at 3 h. (**B**) Exposure of cells to cyanidin (100 μM) decreased reactive oxygen species (ROS) at 3 h of MG incubation. (**C**) Cyanidin (100 μM) alone altered reactive oxygen species (ROS) levels at 3 h of incubation. Results are presented as mean ± SEM (*n* = 3). The groups that do not share a common letter are significantly different (*p* < 0.05).

Treatment of cells with 500 µM MG increased ROS production during a 6 h period (Figure 3A). The greatest increase in ROS was obtained with 3 h incubation. Under this condition, cotreatment with cyanidin (33 and 100 µM) resulted in a 20% and 50% reduction in MG-induced ROS generation (Figure 3B). In addition, 100 µM cyanidin alone caused a decrease (*p* < 0.05) in ROS (Figure 3C).

**Figure 4:**
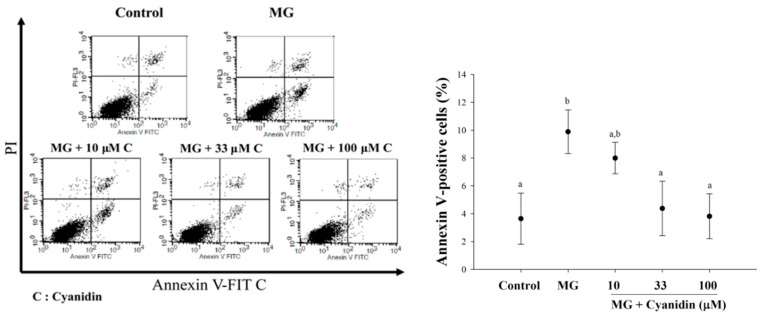
Effect of cotreatment of cyanidin and 500 μM methylglyoxal (MG) for 12 h on apoptosis. Cyanidin (33–100 μM) reduced MG-induced apoptosis in INS-1cells by the PI/Annexin V-FITC stain. Results are presented as mean ± SEM (*n* = 3). The groups that do not share a common letter are significantly different (*p* < 0.05).

Treatment of cells with MG significantly increased the proportion of early apoptotic cells (10%) compared to the controls (Figure 4). Interestingly, cyanidin at 33 and 100 μM concentrations reduced the proportion of early apoptotic cells about 60% and 70%, respectively, when compared to the MG-induced apoptosis.

**Figure 5:**
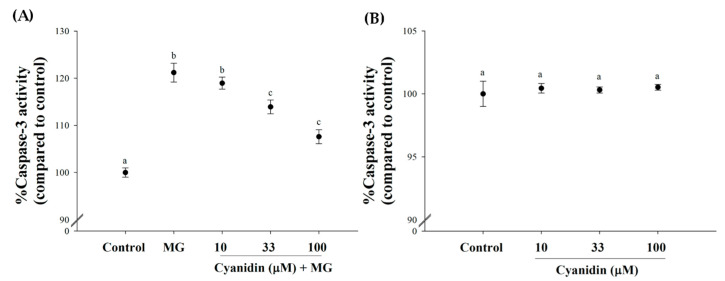
Effect of cotreatment of cyanidin and 500 μM methylglyoxal (MG) for 12 h on caspase-3 activity. (**A**) Cyanidin (33–100 μM) decreased MG-induced increase in caspase-3 activity. (**B**) Cyanidin (10–100 μM) alone had no effect on alteration of caspase-3 activity. Results are presented as mean ± SEM (*n* = 3). The groups that do not share a common letter are significantly different (*p* < 0.05).

In addition, MG induced a 1.21-fold increase in caspase-3 activity, whereas 33–100 μM cyanidin decreased its activity in the range of 7–12%, respectively (Figure 5A). Cyanidin alone failed to activate caspase-3 (Figure 5B)

**Figure 6:**
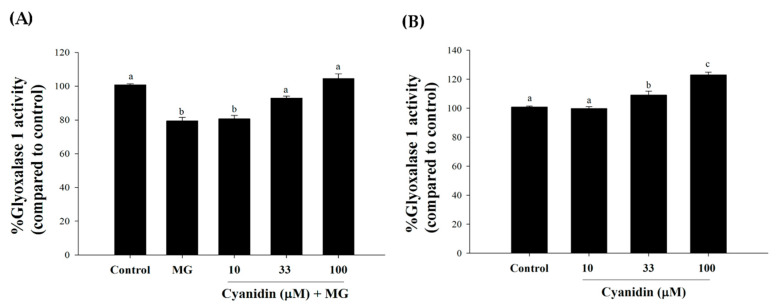
Effect of cotreatment of cyanidin and 500 μM methylglyoxal (MG) for 12 h on glyoxalase 1 (Glo-1) activity. (**A**) Cyanidin (33–100 μM) protected MG-induced decrease in Glo-1 activity. (**B**) Cyanidin (33–100 μM) alone induced an increase in Glo-1 activity. Results are presented as mean ± SEM (*n* = 3). The groups that do not share a common letter are significantly different (*p* < 0.05).

To investigate cyanidin’s protective mechanism against the effects of MG, the activity of glyoxalase 1 (Glo-1) was examined. Treatment of cells with 500 µM MG decreased Glo-1 activity by 22% (*p* < 0.05, Figure 6A). These results demonstrated that cyanidin (33 and 100 µM) maintained Glo-1 activity during MG exposure. In addition, cyanidin at 33 and 100 µM increased Glo-1 activity by 10% and 23%, respectively (Figure 6B). 

**Figure 7:**
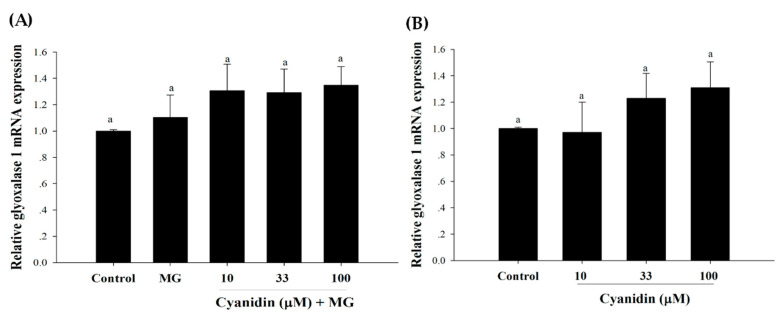
Effect of cotreatment of cyanidin and 500 μM methylglyoxal (MG) for 12 h on the mRNA expression of glyoxalase 1 (Glo-1). (**A**) The mRNA expression of Glo-1 was not reduced by MG or its treatment with cyanidin. (**B**) Cyanidin (10–100 μM) alone had no effect on the mRNA expression of Glo-1. Results are presented as mean ± SEM (*n* = 3). The groups that do not share a common letter are significantly different (*p* < 0.05).

The results revealed that MG did not change the mRNA expression of Glo-1 (Figure 7A,B). In addition, the mRNA expression of Glo-1 was not increased by cyanidin and its cotreatment with MG.

The effect of cyanidin on MG-induced glutathione depletion is shown in Table 1. Cyanidin (10–100 µM) caused a significant increase in glutathione levels and prevented MG-induced depletion.

## 4. Discussion

Numerous studies revealed that MG affects cell viability through oxidative stress-induced cell apoptosis, activates caspases, induces the modification or inactivation of ROS scavenger enzymes, and depletes the cellular glutathione redox status [24,25]. In this regard, MG cytotoxicity is dependent on the exposure time and concentration in pancreatic β-cells [26]. Our results are consistent with findings where MG 500–1000 µM decreases cell viability in concentration and time-dependent manner. This study also demonstrated that MG increased the generation of intracellular ROS and caspase-3 activity, suggesting that MG activates the early apoptotic-signaling pathway in pancreatic β-cells. Excess ROS production is a key factor contributing to mitochondrial apoptosis through caspase-3 activation [27]. Our findings also revealed that cyanidin cotreatment was more effective in maintaining cell viability than pretreatment during exposure to MG. Furthermore, cyanidin cotreatment markedly decreased MG-induced cell apoptosis. The study also found that cyanidin (100 µM) reduced ROS generation in the presence and absence of MG. This suggests that the effect of cyanidin may be related to its free radical and MG scavenging activity, thereby reducing MG-induced ROS generation and promoting a decrease in apoptosis. This observation is supported by findings from Suantawee et al., where cyanidin protects against MG-induced protein glycation and oxidative damage to DNA by trapping reactive dicarbonyl MG [18]. In addition, cyanidin is reported to be an antioxidant that scavenges superoxide and hydroxyl radical generated from the lysine/MG/Cu^2+^ system [28,29]. These effects decrease the level of a secondary product of oxidation malonaldehyde from the degradation of the 2-deoxyribose unit in DNA. Therefore, the superoxide and hydroxyl radical scavenging ability could be responsible for the reduction of MG-induced ROS generation after cyanidin treatment.

The mechanism of the actions of cyanidin described in our study are in accordance with the ones observed for cyanidin-3-rutinoside with a rutinosyl group at the 3-position. It is noteworthy that cyanidin-3-rutinoside acts as an anti-carbonyl stressor agent by trapping free MG and thus, forming C3R-mono-MG adduct [30]. Furthermore, in vivo and in vitro studies suggest that cyanidin-3-rutinoside improves the effects of MG on the vascular system [31,32]. The beneficial effects of cinnamic acid derivatives (isoferulic acid and ferulic acid) on MG-induced apoptosis in INS-1 cells is well established [20,21]. However, the lack of MG-trapping activity of isoferulic acid and ferulic acid is known for the lysine/MG system [20,21]. Based on these observations, the MG trapping capability of cyanidin may not be the only mechanism responsible for protecting pancreatic β-cells from MG-induced apoptosis. 

Several studies show that MG suppresses Glo-1 activity and gene expression in pancreatic β-cells [10,33]. Interestingly, over-expression or increased activity reduces glycation-derived AGEs and MG-induced reactive carbonyl and oxidative stress in mammalian cells [34,35]. Glo-1 converts MG into to S-D-lactoylglutathione by utilizing glutathione, while Glo-2 catalyzes S-D-lactoylglutathione into D-Lactate while regenerating GSH in the process [36]. It is possible that MG induces cellular GSH depletion, resulting in damage to the glyoxalase system. Phytochemical compounds protect against MG-induced damage to pancreatic β-cells by increasing the activity of the MG detoxification system. For instance, sciadopitysin protects against MG-induced cell damage by increasing Glo-1 activity [11]. Enhanced activity is also observed in RIN-5F cells treated with MG and magnolol [37]. Consistent with these studies, we observed a significant decrease in Glo-1 activity and GSH level in INS-1 cells exposed to MG. This effect was reversed by cyanidin without altering gene expression levels. Interestingly, cyanidin alone increased Glo-1 activity and GSH levels in INS-1 cells. It is possible that free radical and MG scavenging of cyanidin may account for the mechanism preventing MG-induced cell damage. This could sustain Glo-1 activity and GSH levels during the action by MG. Studies suggest that Nrf2 activation may regulate Glo-1 activity, thereby protecting pancreatic β-cells from damage [38]. Furthermore, this is evidence for resveratrol regulation of Nrf2 expression with a decrease in MG-induced mitochondrial damage and apoptosis [39]. Moreover, MG-induced ROS generation causes cell apoptosis through activation of ER stress-JNK signaling and mitochondrial pathway [6]. Certainly, further studies are warranted to determine the effect of cyanidin on MG-induced apoptosis through Nrf2 and ER stress-JNK signaling and the mitochondrial pathway in INS-1 cells and animal models. However, the lack of positive control (aminoguanidine) for comparing effectiveness of cyanidin was a limitation of the present study, and additional studies are required to identify this aspect.

## 5. Conclusions

This study provides the first evidence for a protective role of cyanidin during MG-induced oxidative damage and apoptosis in INS-1 cells. These effects could be attributed to the suppression of numerous MG-induced processes (e.g., reduction of ROS and caspase-3 activity). There is now evidence that increased Glo-1 activity and sustained glutathione levels appear part of cyanidin’s mechanism against MG-induced cell toxicity. Taken together, these results suggest that cyanidin could potentially be used to prevent MG-induced oxidative damage and apoptosis in pancreatic β-cells. In addition, cyanidin may be used as a promising agent for functional food and nutraceuticals related to diabetic complications.

## Figures and Tables

**Table 1:** Effect of cyanidin and its cotreatment with 500 μM methylglyoxal (MG) for 12 h on total glutathione.

Treatments	Total Glutathione (nmol/mg protein)
Control	3.49 ± 0.13 ^a^
500 μM MG	1.66 ± 0.05 ^b^
500 μM MG + 10 μM cyanidin	4.03 ± 0.28 ^a^
500 μM MG + 33 μM cyanidin	4.96 ± 0.54 ^a^
500 μM MG + 100 μM cyanidin	6.35 ± 0.31 ^c^
10 μM cyanidin	5.37 ± 0.73 ^c^
33 μM cyanidin	6.28 ± 0.90 ^c^
100 μM cyanidin	7.15 ± 0.77 ^d^

Results are presented as mean ± SEM (*n* = 3). The groups that do not share a common letter are significantly different (*p* < 0.05).
